# Evidence on novel endovascular techniques in the ESO/EANS/ESMINT guideline on aneurysmal subarachnoid haemorrhage

**DOI:** 10.1093/esj/aakag074

**Published:** 2026-07-08

**Authors:** Markus A Möhlenbruch, Martin Bendszus

**Affiliations:** Department of Neuroradiology, Heidelberg University Hospital, Im Neuenheimer Feld 400, Heidelberg 69120, Germany; Department of Neuroradiology, Heidelberg University Hospital, Im Neuenheimer Feld 400, Heidelberg 69120, Germany

**Keywords:** endovascular techniques, ESO/EANS/ESMINT guideline, ruptured aneurysms

Dear Kennedy Lees,

We read with interest the recent ESO/EANS/ESMINT guideline on the management of ruptured intracranial aneurysms by Vergouwen et al. and would like to respectfully comment on the interpretation of contemporary endovascular treatment strategies—specifically balloon-assisted coiling (BAC) and intrasaccular flow disruption devices.^1^ In addition, relevant differences to the most recent American Heart Association/American Stroke Association (AHA/ASA) guideline warrant consideration in this context. In contrast to earlier guideline iterations, the current AHA/ASA guideline explicitly reflects the evolution of endovascular techniques beyond coiling alone, incorporating adjunctive and device-assisted strategies within an individualised treatment framework.^2^

Aneurysm treatment evidence spans multiple technological generations, and failure to distinguish early feasibility-era data from contemporary multicentre evidence may lead to indirect comparisons that underestimate current endovascular safety and efficacy.

## Balloon-assisted coiling (BAC)

Balloon-assisted coiling is an established and widely used adjunct for wide-neck ruptured intracranial aneurysms, thereby avoiding the need for a permanent implant and prolonged antiplatelet therapy.

Contemporary multicentre data, registries and systematic reviews consistently demonstrate that BAC is both safe and effective, with high technical success rates and acceptable periprocedural complication profiles.^3^ In addition, the prospective multicentre CLARITY study demonstrated higher angiographic occlusion rates with BAC compared with conventional coiling, while overall treatment-related complication rates remained comparable. This contemporary dataset is cited in the guideline, yet its implications for contemporary BAC practice are not extensively discussed.

In contrast, parts of the evidence cited against BAC in the guideline (notably refs 48–50, including one letter to the editor) originate from 2 early single-centre experiences performed during the initial adoption phase of BAC. These studies are characterised by limited sample sizes, substantial selection bias, heterogeneous procedural protocols and early-generation devices. Such methodological limitations reduce the applicability of these early experiences to current BAC practice using modern devices and standardised workflows.

## Intrasaccular flow disruption devices

Intrasaccular flow disruption devices represent a more recent evolution in endovascular aneurysm therapy. The prospective multicentre CLARYS study reported low periprocedural complication rates without WEB-related morbidity or mortality at 1 month or 1 year. In addition, the guideline cites a relevant meta-analysis comparing the Woven EndoBridge (WEB) device to stent-assisted and conventional coiling across 16 studies published before 2024.^4^ In this analysis, WEB treatment was associated with lower complication rates while maintaining favourable angiographic and clinical outcomes. In contrast to the statement in the current guideline, the core-lab-assessed WEB-IT study and CLARYS showed a favourable safety and efficacy profile in the treatment of cerebral aneurysms. These contemporary prospective and pooled data suggest that intrasaccular flow disruption devices should be interpreted within the context of current endovascular practice rather than extrapolated from earlier coiling-era experience.

## Microsurgical clipping

Microsurgical clipping has historically been established on the basis of large institutional experiences and observational surgical series, reflecting the practical challenges of randomised evaluation in cerebrovascular surgery.^5^ In contrast, contemporary endovascular technologies—particularly novel adjunctive and device-based approaches—are increasingly introduced within prospective multicentre registries under Good Clinical Practice conditions and formalised comparative study frameworks prior to broad clinical adoption. These differences in evidence generation pathways and evidentiary expectations should be considered when interpreting comparative recommendations across treatment modalities.

## Interpretation of evidence across modalities

Overall, the evidence base underlying comparative statements in the guideline spans markedly different treatment eras and levels of methodological rigour.

Importantly, more recent prospective multicentre data on BAC and intrasaccular flow disruption devices demonstrate consistently high occlusion rates and favourable clinical outcomes (CLARITY, CLARYS, WEB-IT). Taken together, these contemporary prospective and pooled datasets should not be interpreted as direct comparative evidence against ISAT-era coiling, given substantial differences in patient selection, device technology and periprocedural management. Nevertheless, they demonstrate that the technological and procedural landscape of endovascular aneurysm therapy has evolved substantially beyond the early coil embolisation era reflected in ISAT. Consequently, the applicability of historical coiling-era outcomes for evaluating current endovascular device performance may be limited. A structured stratification of evidence according to the study era, device generation and methodological quality could improve interpretability and better align guideline recommendations with contemporary practice.

## Conclusion

We respectfully suggest that future guideline updates may benefit from a more granular differentiation of evidence according to methodological quality, study era and device generation. This is particularly relevant for BAC and intrasaccular flow disruption techniques, where substantial technological evolution has significantly altered procedural safety and efficacy profiles.

Such refinement would strengthen the translational relevance of guideline recommendations in contemporary neurovascular practice and better support individualised, multidisciplinary decision-making for patients with ruptured intracranial aneurysms.
